# C-Reactive Protein (CRP) Blocks the Desensitization of Agonistic Stimulated G Protein Coupled Receptors (GPCRs) in Neonatal Rat Cardiomyocytes

**DOI:** 10.3390/jcm11041058

**Published:** 2022-02-17

**Authors:** Gerd Wallukat, Stephan Mattecka, Katrin Wenzel, Wieland Schrödl, Birgit Vogt, Patrizia Brunner, Ahmed Sheriff, Rudolf Kunze

**Affiliations:** 1 Berlin Cures GmbH, BBB Campus, 13125 Berlin, Germany; gwalluk@berlincures.de (G.W.); kwenzel@berlincures.de (K.W.); 2 Pentracor GmbH, 16761 Hennigsdorf, Germany; mattecka@pentracor.de (S.M.); vogt@pentracor.de (B.V.); brunner@pentracor.de (P.B.); ahmed.sheriff@charite.de (A.S.); 3 Institute of Bacteriology and Mycology Faculty of Veterinary Medicine, University of Leipzig, 04103 Leipzig, Germany; schroedl@rz.uni-leipzig.de; 4 Division of Gastroenterology, Infectiology and Rheumatology, Medical Department, Charité University Medicine, 12200 Berlin, Germany

**Keywords:** C-reactive protein, adrenergic receptor, desensitization, GPCR signaling, endothelin

## Abstract

Recently, C-reactive protein (CRP) was shown to affect intracellular calcium signaling and blood pressure in vitro and in vivo, respectively. The aim of the present study was to further investigate if a direct effect on G-protein coupled receptor (GPCR) signaling by CRP can be observed by using CRP in combination with different GPCR agonists on spontaneously beating cultured neonatal rat cardiomyocytes. All used agonists (isoprenaline, clenbuterol, phenylephrine, angiotensin II and endothelin 1) affected the beat rate of cardiomyocytes significantly and after washing them out and re-stimulation the cells developed a pronounced desensitization of the corresponding receptors. CRP did not affect the basal beating-rate nor the initial increase/decrease in beat-rate triggered by different agonists. However, CRP co-incubated cells did not exhibit desensitization of the respective GPCRs after the stimulation with the different agonists. This lack of desensitization was independent of the GPCR type, but it was dependent on the CRP concentration. Therefore, CRP interferes with the desensitization of GPCRs and has to be considered as a novel regulator of adrenergic, angiotensin-1 and endothelin receptors.

## 1. Introduction

The homopentameric C-reactive protein (CRP) is a classic acute phase protein that has been known in human medicine for decades. It has been established primarily as a biomarker for active and chronic inflammation of bacterial origin [1] This picture has changed fundamentally. In the 1990s, CRP was identified as a risk factor for atherosclerosis. Relatively low blood levels at >2 mg/L CRP are associated with an increased risk of heart attack, stroke, diabetes and mortality depending on the concentration that was observed [2,3,4]. Acute inflammation caused by vessel occlusions can be observed in acute myocardial infarction, with rapidly increasing CRP levels up to >100 mg/L over 2–4 days. Restrictions of organic functions may be the consequences of CRP mediated ischemic processes [5,6].

Publications in recent years showed that CRP is more than a biomarker and affects both physiological and pathological processes [5,7,8,9]. The direct influence of CRP on the cardiovascular system of rabbits has been reported recently [10]. Here, the infusion of human CRP led to a sharp drop in blood pressure within seconds, while the heart rate was not affected. The authors also investigated the influence of CRP on calcium signaling in vitro in two epithelial cell lines. The activation of the cells by adrenoceptor agonists led to intracellular Ca^2+^ mobilization, which was further increased in the presence of CRP [10].

CRP progressively emerges as a molecule with regulatory properties besides its role as an acute phase molecule. In the case of acute inflammation and the associated high CRP levels in the bloodstream, this pentamer primarily has contact with mobile leukocytes and sessile cells lining the vessels, especially endothelial cells. Although interaction with receptors on these cells seems obvious, CRPs molecular action has so far only been investigated on, e.g., Fc receptor γRII (FcγRII) and in the context of macrophage activation and its role as an archaic antibody-like molecule [11,12,13]. However, one of the fundamental roles of receptor signaling in endothelial cells is the regulation of circulatory parameters, which is mainly mediated by G protein-coupled receptors (GPCRs), specifically adrenergic receptors, angiotensin-II receptor (AT1) and endothelin receptors (ETRs) [14]. Moreover, this is of great impact during excessive inflammatory states, such as, e.g., septic shock, in which circulating CRP levels are dramatically high and hemodynamic parameters are considerably unstable [15,16].

In this respect, it is obvious to examine the influence of CRP on receptor-controlled cell-physiological activation processes. In this paper, we report on the influence of CRP on the signaling system of selected GPCRs, which are involved in the regulation of cardiac, smooth or skeletal muscle cells.

GPCRs are a large group of membrane-bound proteins, the amino acid chain of which crosses the cell membrane seven times. The group is named after the receptor activation triggered extracellularly by agonists, which leads to an interaction with G proteins intracellularly [17]. Adrenoceptors can be classified into five groups (α1, α2 and β1, β2, β3) and are expressed more or less in different tissues and organs of the body [18,19]. They are primarily activated by catecholamines and generally affect the contraction of smooth muscle cells, thereby fundamentally regulating the heart rate and blood pressure. Physiologically important agonists are noradrenaline or adrenaline. Pharmacological, synthetic receptor-specific agonists, such as isoprenaline (β1 and β2-adrenoceptor), clenbuterol (β2-adrenoceptor) or phenylephrine (α1-adrenoceptor), are drugs that are frequently used in pharmacological experiments [14,19,20]. In addition, the peptide hormones endothelin 1 or angiotensin II activate the endothelin ETA and ETB-receptor and the angiotensin II AT1 and AT2-receptor, respectively.

The β1-adrenoceptor is the major adrenergic receptor of the myocardium. Besides this receptor, the β2 and β3 adrenoceptors and the α1-adrenergic, angiotensin II AT1- and endothelin 1 ETA receptors are also expressed in this organ. This receptor expression can be modulated by a long-term treatment with the corresponding agonist and antagonists and also by the agonistic autoantibodies [21,22,23,24].

The AT1-, ETA- and α1 receptors are receptors of the blood vessels and are known as vasoconstrictors [25]. However, other receptors such as the β2 adrenoceptor are also expressed in the vasculature. Our investigation has shown that cultured rat cardiomyocytes express a multitude of different G-protein coupled receptors that are coupled to different signal cascades and can change the beating rate of the used spontaneously beating cardiomyocytes [26].

The physiological effect of receptor agonists has been studied for decades on the cultures of neonatal rat cardiomyocytes. These cells beat spontaneously in culture and the change in the pulsation rate after the addition of the agonists can be measured visually on an inverted microscope. This bioassay is a standard cell biological method for the identification and characterization of functional autoantibodies against GPCR or the effect of pharmacological receptor antagonists [27,28].

The aim of the study was to investigate if the inflammatory acute phase protein CRP interferes with the regulation of GPCRs on the cellular level. Here we present first observations about the inhibition of the elementary process of receptor desensitization in rat cardiomyocytes.

## 2. Materials and Methods

### 2.1. Pharmacological Agonists and CRP

Human CRP (Pentracor, Hennigsdorf, Germany) was purified from human pooled plasma with a selective CRP-binding matrix as described elsewhere in detail [10]. Endotoxin contamination was avoided, and CRP was stored in its native, pentameric form.

The murine monoclonal antibody (0.64 mg/mL) against CRP was provided by Biometec Inc. (Dr. S. Witt, Biometec GmbH, Greifswald, Germany) and generated by Dr. B. Micheel and Dr. W. Schroedl.

The pharmacological agonists were purchased from Sigma, Germany (Isoprenaline, Phenlyephrine, Endothelin 1, Clenbuterol) or MP Biomedicals, Germany (Angiotensin II) and used in concentrations indicated in Table 1.

### 2.2. Cardiomyocyte Bioassay

The cardiomyocyte bioassay was carried out on neonatal rat cardiomyocytes in cell culture as described elsewhere in detail [27]. Briefly, cardiac myocytes were prepared from heart ventricles of 1–2-day-old Sprague-Dawley rats and cultured in SM20-I medium (Biochrom, Berlin, Germany) with supplemented penicillin (Heyl, Berlin, Germany), heat-inactivated 10% neonatal calf serum (Gibco, Life Technologies, Bleiswijk, The Netherlands), glutamine (Serva, Heidelberg, Germany), streptomycin (HEFA Pharma; Werne, Germany), hydrocortisone (Merck, Darmstadt, Germany) and fluorodeoxyuridine (Serva, Heidelberg, Germany). After seeding the cardiomyocytes with a field density of 160,000 cells/cm^2^, the culture medium was renewed after 24 h. The cells were cultured for 2–4 days at 37 °C before using them in the experiment. Target point was the beating rate for 15 s of 6–10 synchronously contracting cell clusters per flask, placed on a heated stage of an inverted microscope at 37 °C. First, the basal beating rate of the cardiomyocytes was measured and after this the agonists were added. The difference between the basal beating rate and increase or decrease in the beating rate after the addition of the agonists is expressed as Δ beating rate/15 s. Respective agonists were then added to the cell culture medium and beating rate was measured again after 5 and 120 min. This was followed by a change of medium (washing 3 times with warm PBS (without calcium), then adding fresh prewarmed (37 °C) complete SM20 culture medium. Thereafter, the pulsation rate returned to the initial value (measured at t = 125 min). After this the stimulation with the agonists was repeated in the same agonist concentration.

For CRP co-incubated cells, human CRP was added in a final concentration of either 50 µg/mL or in decreasing concentrations of 40, 20, 10, 5 and 2 µg/mL and preincubated 10 min before agonist stimulation. CRP was not added again after 125 min.

To block the activity of CRP, it was pretreated with a blocking monoclonal antibody (0.64 mg/mL) directed against CRP for 30 min at room temperature. This mixture was added to the cardiomyocytes like the CRP solution as described above.

## 3. Results

**Figure 1:**
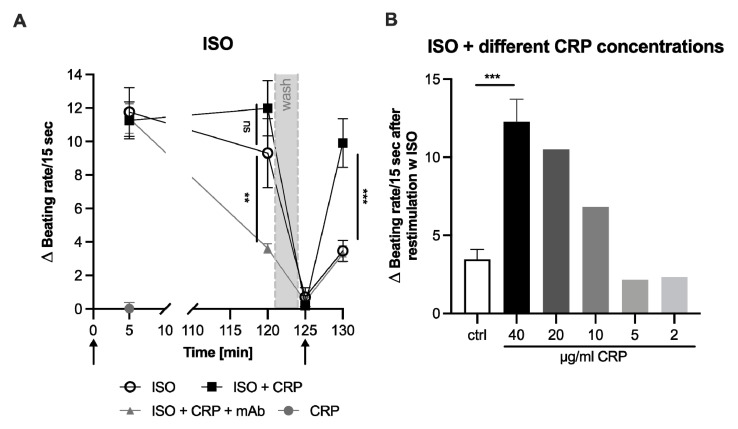
CRP blocks the desensitization of stimulated neonatal rat cardiomyocytes with ISO. Difference of cardiomyocyte beating rates [Δ Beating rate/15 s] modulated by adrenoceptor agonist isoprenaline (ISO) with or without CRP co-incubation. (**A**) Mean of 3–7 independent experiments ± standard deviation is depicted. Arrows indicate stimulation with respective agonists. Grey area indicates washing step. After the washing step, the agonist was added again but no CRP. To test if CRP itself influences the beating rate, cells were incubated with CRP and no change could be observed after 5 min (grey circle). Significance of difference between CRP incubated and control group and mAb group at 120 min and 130 min was calculated with two-way ANOVA with Bonferroni post-hoc test. (**B**) Same experimental setup as depicted in (**A**), but only the beating rate at the last time point (130 min) is shown as mean of 3–5 independent experiments ± standard deviation or as single value (20–2 µg/mL CRP). Significance of difference between 40 µg/mL CRP and control (only ISO) was calculated with student’s *t*-test. ** *p* < 0.01. *** *p* < 0.001. ns: not significant.

First, cardiomyocytes were stimulated with isoprenaline (ISO) with and without CRP for 120 min. As expected, ISO increased the beating frequency compared to the basal rate (0; Figure 1A). The initial beating rate was not affected by CRP addition (5 min). After 120 min, cells were washed and the beating rates returned to their basal rate (Figure 1A, 125 min). Then, cells were stimulated again with ISO. In control cells (without CRP), the increase in the pulsation rate was clearly diminished after the renewed stimulation with ISO (Figure 1A, 130 min). This can be explained by the desensitization of adrenergic receptors and was expected [29]. However, with additional CRP incubation, the cardiomyocyte beating rate increased to a similar level compared to the initial stimulation and was significantly higher compared to the control experiment. No desensitization of the response was visible in CRP co-incubated cells. Cells that were treated with CRP preincubated with a monoclonal antibody (mAb) to inhibit the CRP action showed normal desensitization and a beating rate comparable to that in ISO stimulated cells alone after 130 min (Figure 1A). Further, this blocking of the desensitization was concentration dependent. CRP was applied in decreasing final concentrations (40–2 µg/mL) and the same experimental setup was repeated. CRP blocked desensitization after 2nd ISO stimulation in a concentration dependent manner (Figure 1B).

**Figure 2:**
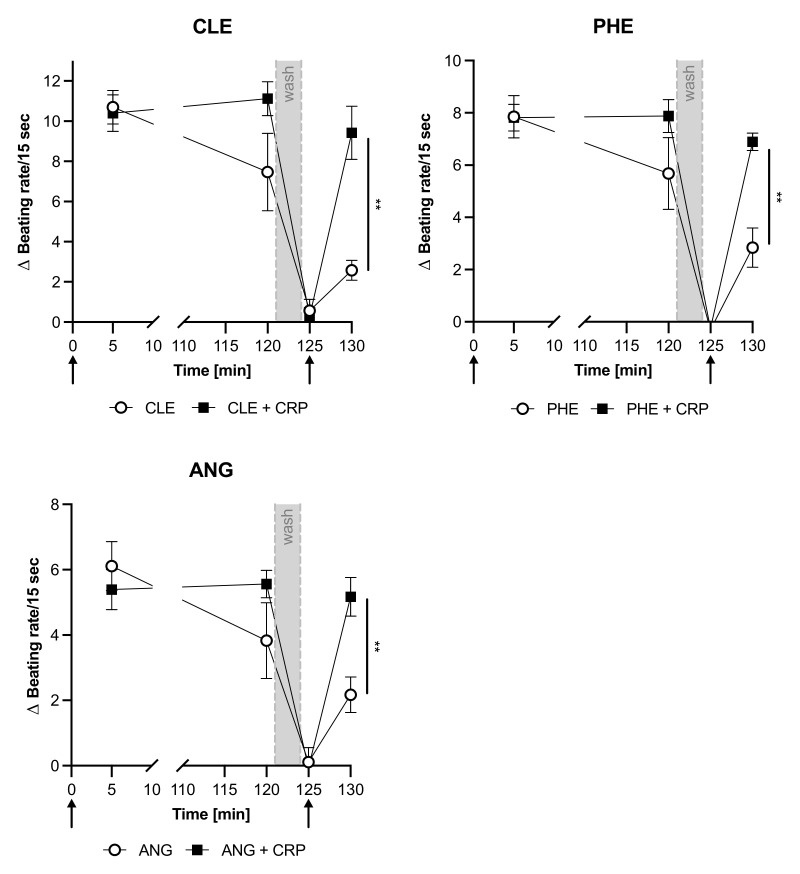
CRP blocks the desensitization of stimulated neonatal rat cardiomyocytes. Difference of cardiomyocyte beating rates [Δ Beating rate/15 s] modulated by agonists clenbuterol (CLE), phenylephrine (PHE) and angiotensin II (ANG) with or without CRP co-incubation. Mean of 3–5 independent experiments ± standard deviation is depicted. Arrows indicate stimulation with respective agonists. Grey area indicates washing step. After the washing step, the agonist was added again but no CRP. Significance of difference between CRP incubated and control group at the last time point was calculated with student’s *t*-test. ** *p* < 0.01.

This CRP-induced prevention of desensitization was not only seen for the β-adrenoceptors but also for other GPCRs. In our experiments, we additionally tested the effect of CRP on the β2-adrenoceptor (stimulated with clenbuterol (CLE)), the α1-adrenoceptor (stimulated with phenylephrine (PHE)), and the angiotensin II AT1 receptor (stimulated with angiotensin II (ANG)). These receptor agonists also exert a positive chronotropic response and the desensitization of these GPCR was also prevented by CRP (Figure 2). Although phenylephrine and angiotensin II are not highly specific for the indicated receptors, the response can be attributed to these. Only blocking with specific antagonists against these receptors inhibits the chronotropic response in the bioassay [30,31].

To investigate whether this was exclusive to positive chronotropic agonists that realize their effects via the α1-, β1-, β2- adrenoceptors or the AT1-receptor, cells were also stimulated with endothelin 1 (ET-1), which binds to ETRs and exerts a negative chronotropic effect in the spontaneously beating rat cardiomyocytes.

**Figure 3:**
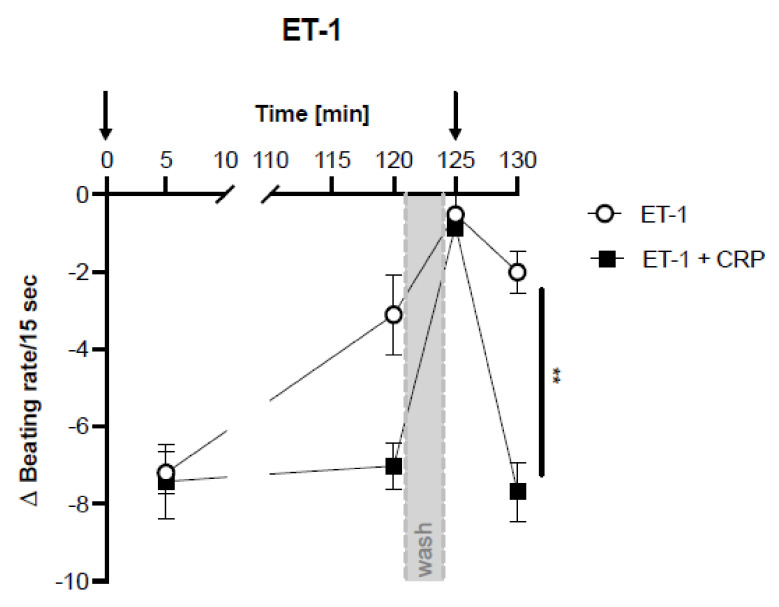
CRP blocks the desensitization of endothelin stimulated neonatal rat cardiomyocytes. Difference of cardiomyocyte beating rates [Δ Beating rate/15 s] modulated by endothelin receptor agonist endothelin 1 (ET−1) with or without CRP co-incubation. ET1 induced a negative chronotropic response. Mean of 4 (control) or 3 (with CRP) independent experiments ± standard deviation is depicted. Arrows indicate stimulation with respective agonists. Grey area indicates washing step. After the washing step, the agonist was added again but no CRP. Significance of difference between CRP incubated and control group at the last time point was calculated with student’s *t*-test. ** *p* < 0.01.

ET-1 decreased the cardiomyocyte beating rate initially with and without CRP (Figure 3). Again, responsive receptors were desensitized after 120 min of incubation and after washing and renewed stimulation with ET-1, visible by only slightly decreased beating rates at 120 and 130 min (Figure 3). Co-incubation with CRP abolished the desensitization effect and beating rates were again significantly reduced after renewed ET-1 stimulation.

CRP stimulation alone did not affect the cardiomyocyte beating rate as visible in Figure 1A after 5 min (Δ beating rate/15 s = 0.02 (*n* = 7)).

Detailed data values of all replicate experiments are listed in Table 1.

## 4. Discussion

This study was designed to further investigate possible direct effects of CRP on GPCR signaling. Therefore, an established in vitro model was used, spontaneously beating neonatal rat cardiomyocytes, which react to stimulations with agonists or antagonists of different GPCRs with a change of beating rates [32].

Stimulation of cardiomyocytes with different adrenergic, AT-1 and ETR agonists readily affected their beating rate as expected. Co-incubation with CRP did not influence the basal beating rate nor the initial effect of the agonistic stimulation. However, desensitization of GPCRs, which was observed with all used agonists, did not occur in CRP co-incubated cardiomyocytes (Figure 1A, Figure 2 and Figure 3, 130 min). Re-stimulation with either isoprenaline, clenbuterol, phenylephrine, angiotensin or endothelin-1 showed a significant chronotropic effect in CRP co-incubated cells, on a similar level as the initial stimulation. This was already visible as a trend after 120 min, when CRP-co-incubated cells showed slightly higher beating rates than agonist-only treated cells (Figure 1A, Figure 2 and Figure 3, 120 min), albeit not significant. CRP pretreated with a blocking monoclonal antibody, blocked the effect seen in the presence of CRP and led to the desensitization of the receptor-mediated response. Surprisingly, the antibody influenced the beating rate after 120 min and led to a significant reduction compared to cells treated only with isoprenaline or with isoprenaline and CRP (Figure 1A, 120 min). Fetal calf serum has been shown to contain CRP [33]. It cannot be excluded that this already has an effect, although the neonatal calf serum has been heat inactivated. Since CRP was not suspected of influencing this test system, this issue has not yet been investigated.

This leads to the conclusion that this acute phase protein somehow inhibits the mechanism of desensitization. This phenomenon, which is the same in all GPCRs examined, suggests ubiquitous mechanisms that take place via the cell membrane and therefore, potentially affect all GPCRs.

GPCR signaling is naturally regulated in a highly complex manner and on several levels. Desensitization is a basic physiological principle, employed by cells in order to protect themselves from overstimulation and possible exhaustion. Receptors can be rendered unresponsive by sequestering or degrading them or downstream intracellular messengers. Rapid desensitization, which would be the case in our applied experimental timeline, is mainly achieved by GPCR phosphorylation, uncoupling the receptor from its respective G protein [29]. This phosphorylation is mostly mediated by GPCR kinases, leading to binding of arrestins, which block further signaling [34].

The blockade of desensitization of the examined GPCRs achieved by CRP goes beyond the known inflammatory properties of CRP. To hypothesize which molecular route CRP modulates in order to produce a second chronotropic reaction to GPCR agonists would be purely speculative and cannot be deduced from these results. It is, however, in line with previous findings, showing a direct effect of CRP on intracellular calcium signaling, which was further increased by ISO or PE stimulation, no matter the order of stimulation [10]. Interestingly, in this experimental setup, when the used GPCR agonist was washed away after 120 min, CRP in the culture medium was also washed off and not reapplied. Hence, the observed effects stem from the pre-incubation of cells with CRP. This means that either CRP is still bound to the cell membrane after washing or already modulated intracellular components, thereby affecting GPCR desensitization. Although this would be a novel action of CRP, it has been previously reported that CRP can either directly stimulate other receptors than Fcγ on cells [35,36] or regulate their expression in vascular smooth muscle cells [37]. This already indicated that the physiological function of CRP is far more complex than assumed.

Obviously, the protective mechanism of the fast desensitization of GPCRs is inhibited by an elevated level of CRP in vitro, with possible pathophysiological consequences in vivo. Hemodynamic parameters are often unstable in extreme inflammatory settings such as sepsis, which also present dramatically high circulating CRP levels with peaks of ≥150 mg/L CRP [15,16]. Although no direct effect of CRP on hemodynamic variables has been shown so far in CRP-infused humans, this is most likely because the published experimental setups have focused on long-term effects or rather low CRP concentrations [38,39,40]. The recently published results in rabbits [10] are in line with the here described effects, which hint towards a process in which CRP itself affects blood pressure and heart rate by modulating complex GPCR signaling in endothelial cells and/or cardiomyocytes.

In conclusion, the acute phase protein CRP may play an important role in the regulation of GPCR signaling. By blocking the desensitization of different GPCRs, CRP—in combination with the corresponding agonist—induces a permanent receptor stimulation that may represent a dangerous pathogenic factor. This permanent stimulation of cells could induce a calcium overload, apoptosis and subsequent cell death. Investigations could be conducted to determine whether this effect on the GPCR regulation could play an additional role in the context of the CRP-mediated tissue destruction during ischemic processes [5]. Therefore, it may be meaningful to remove the elevated CRP levels not only after myocardial infarction [6] but also in, for example, sepsis.

The findings described herein should be a springboard for more elaborate experiments to characterize and understand the molecular details of CRP-mediated inhibition of GPCR desensitization.

### Study Limitations

The studies were carried out on primary neonatal rat cardiomyocytes. Their cell-specific receptor equipment is to be seen primarily as a signaling system. Whether this phenomenon also occurs in other cell types such as endothelial cells was not investigated. Other test systems such as calcium signaling are suitable for this. It should also be a focus to find out more about the molecular mechanism of receptor desensitization including whether CRP directly interacts with the respective GPCRs or other receptors on the cardiomyocytes. If similar data are obtained this way, then the conclusions on the pathophysiological effects of CRP on the homeostasis of GPCR could be further elaborated.

## Figures and Tables

**Table 1:** Effect of CRP on the beating rate of neonatal rat cardiomyocytes stimulated with agonists against GPCRs. The data show the increase or decrease in the beating rate as mean ± standard deviation of the rat cardiomyocytes.

Agonist	*n*	CRP (50 μg/mL)	Difference in Beating Rate/15 s at Incubation Time (min)				*p*-Value
			5	120	125	130	130 min, ±CRP
Isoprenaline (1 μM)	5	−	11.76 ± 1.46	9.31 ± 2.07	0.71 ± 0.56	3.47 ± 0.63	<0.001
	5	+	11.26 ± 1.10	11.99 ± 1.66	0.19 ± 0.61	9.91 ± 1.46	
Clenbuterol (3 μM)	4	−	10.69 ± 0.84	7.47 ± 1.93	0.56 ± 0.57	2.58 ± 0.50	0.001
	4	+	10.40 ± 0.91	11.12 ± 0.85	0.28 ± 0.44	9.42 ± 1.33	
Phenylephrine (10 μM)	3	−	7.85 ± 0.81	5.68 ± 1.38	−0.32 ± 0.43	2.84 ± 0.75	0.006
	3	+	7.82 ± 0.51	7.88 ± 0.63	−0.27 ± 0.17	6.89 ± 0.34	
Angiotensin II (1 μM)	3	−	6.11 ± 0.75	3.83 ± 1.16	0.11 ± 0.44	2.17 ± 0.54	0.006
	3	+	5.39 ± 0.61	5.56 ± 0.42	−0.06 ± 0.08	5.17 ± 0.59	
Endothelin 1 (0,1 μM)	4	−	−7.20 ± 0.55	−4.08 ± 1.91	−0.52 ± 0.53	−2.01 ± 0.53	0.002
	3	+	−7.42 ± 0.96	−7.02 ± 0.60	−0.83 ± 0.24	−7.69 ± 0.76	

## Data Availability

The original contributions presented in the study are included in the article, further inquiries can be directed to the corresponding author.
